# Successful field trial of attractive toxic sugar bait (ATSB) plant-spraying methods against malaria vectors in the *Anopheles gambiae *complex in Mali, West Africa

**DOI:** 10.1186/1475-2875-9-210

**Published:** 2010-07-21

**Authors:** Günter C Müller, John C Beier, Sekou F Traore, Mahamadou B Toure, Mohamed M Traore, Sekou Bah, Seydou Doumbia, Yosef Schlein

**Affiliations:** 1 Hebrew University, Hadassah Medical School, Kuvin Center for the Study of Tropical and Infectious Diseases, Department of Parasitology, Jerusalem, 91120, Israel; 2 Center for Global Health Sciences, Department of Epidemiology and Public Health, University of Miami Miller School of Medicine, Miami, Florida, 33136, USA; 3 Malaria Research and Training Center, Faculty of Medicine, Pharmacy and Odontostomatology, University of Bamako, BP 1805, Bamako, Mali; 4 Department of Pharmaceutical Sciences, Faculty of Medicine, Pharmacy and Odontostomatology, University of Bamako, BP 1805, Bamako, Mali

## Abstract

**Background:**

Based on highly successful demonstrations in Israel that attractive toxic sugar bait (ATSB) methods can decimate local populations of mosquitoes, this study determined the effectiveness of ATSB methods for malaria vector control in the semi-arid Bandiagara District of Mali, West Africa.

**Methods:**

Control and treatment sites, selected along a road that connects villages, contained man-made ponds that were the primary larval habitats of *Anopheles gambiae *and *Anopheles arabiensis*. Guava and honey melons, two local fruits shown to be attractive to *An. gambiae *s.l., were used to prepare solutions of Attractive Sugar Bait (ASB) and ATSB that additionally contained boric acid as an oral insecticide. Both included a color dye marker to facilitate determination of mosquitoes feeding on the solutions. The trial was conducted over a 38-day period, using CDC light traps to monitor mosquito populations. On day 8, ASB solution in the control site and ATSB solution in the treatment site were sprayed using a hand-pump on patches of vegetation. Samples of female mosquitoes were age-graded to determine the impact of ATSB treatment on vector longevity.

**Results:**

Immediately after spraying ATSB in the treatment site, the relative abundance of female and male *An. gambiae *s.l. declined about 90% from pre-treatment levels and remained low. In the treatment site, most females remaining after ATSB treatment had not completed a single gonotrophic cycle, and only 6% had completed three or more gonotrophic cycles compared with 37% pre-treatment. In the control site sprayed with ASB (without toxin), the proportion of females completing three or more gonotrophic cycles increased from 28.5% pre-treatment to 47.5% post-treatment. In the control site, detection of dye marker in over half of the females and males provided direct evidence that the mosquitoes were feeding on the sprayed solutions.

**Conclusion:**

This study in Mali shows that even a single application of ATSB can substantially decrease malaria vector population densities and longevity. It is likely that ATSB methods can be used as a new powerful tool for the control of malaria vectors, particularly since this approach is highly effective for mosquito control, technologically simple, inexpensive, and environmentally safe.

## Background

One of the key challenges for successful malaria control and eventual malaria elimination in African countries is to implement highly efficient malaria vector control to reduce annual entomological inoculation rates (EIRs) to below one infective bite per person per year [1]. Such reductions are required to drive down levels of malaria prevalence to achieve local malaria elimination [2]. Currently, there are no documented examples anywhere in Africa where annual EIRs have been reduced and sustained to levels < 1 using available vector control tools [3].

Current options for vector control tools to tackle the malaria problems in African countries are limited. Most national malaria control programmes use long-lasting insecticide-treated nets (LLINs) and/or indoor residual spraying (IRS) [4,5], and there is a growing interest in environmental management and larval control [6-8]. These proven methods can reduce malaria parasite transmission by > 90%, and correspondingly reduce the incidence of new infections and malaria-related mortality. However, they do not consistently reduce malaria prevalence because even barely detectable low numbers of infective bites per person per year can be associated with malaria prevalence rates over 20% [1]. The lack of viable new methods for vector control is one reason why integrated vector management (IVM) strategies [9,10] have not been fully embraced and implemented [11]. Clearly, new vector control tools that can be used in conjunction with current methods are required as successful malaria control programmes transition their goals to country-wide malaria elimination [5].

This paper addresses whether newly developed ATSB methods may be suitable for malaria vector control in Africa. The ATSB methods, developed and tested extensively in Israel [12-16] represent a new form of mosquito control based on an "attract and kill" principle. The ATSB approach uses fruit or flower scent as an attractant, sugar solution as a feeding stimulant, and oral toxin to kill the mosquitoes. The ATSB solutions are either sprayed on vegetation or suspended in simple bait stations, and the mosquitoes ingesting the toxic solutions are killed. As such, this method targets sugar-feeding female and male mosquitoes outdoors. Plant sugars or "sugar meals" represent an important source of energy for female mosquitoes and are the only food source for males [17,18]. Data on mosquito orientation to plant volatiles and their attraction to plant odors were recently reviewed [19]. Over a range of arid environments in Israel, field trials of ATSB methods have proven highly effective in decimating local populations of diverse mosquito species. In addition to being highly effective, technologically simple, and low-cost, the ATSB methods are based on the use of oral toxins as opposed to contact insecticides used in LLINs or IRS. As such, this new approach circumvents many of the traditional problems relating excito-repellency and the development of insecticide resistance in mosquitoes [20,21].

The objective of this study was to conduct a controlled field trial of ATSB plant-spraying methods to determine impact on malaria vector densities and longevity in a semi-arid malaria endemic area of Mali. This study represents the first evaluation of ATSB methods for malaria vector control in Africa.

## Methods

### Study sites

The study was conducted at the margins of the inland delta of the river Niger in Bandiagara District, approximately 650 km northeast of Bamako, Mali. In this semi-arid area, the rainy season is between July and September with a peak of malaria transmission in October. Malaria vectors include 99.8% *Anopheles gambiae *s.l., of which 86% are *An. gambiae *s.s and 14% are *Anopheles arabiensis *and *Anopheles funestus *[22]. Malaria transmission is seasonal with virtually undetectable transmission during the dry season and up to 25 infective bites per person per month during peak periods of transmission. The prevalence of *Plasmodium falciparun *infection varies from 45% during the dry season to > 65% at the end of the rainy season [23].

An area along the main road, connecting Bamako and Gao, about 50 km north of Sevare provided ideal testing conditions for an ATSB field trial, in terms of both representative local environmental conditions and relatively isolated ecological "island" settings with abundant larval habitat containing high densities of *An. gambiae *s.l. The area contains numerous clusters of three to five ponds for collecting rainwater with the ponds varying in size from 3,000 to > 10,000 square meters. They were artificially created to assist the semi-nomadic population during the dry season and are used as a water supply for local livestock and the shallow areas for rice paddies. The clusters of ponds are separated from each other by 0.5 to 3 km and are surrounded by arid vegetation. Larval surveys conducted in seven clusters of ponds along a road that interconnects local villages showed that most of the ponds contained *An. gambiae *s.l. larvae. From the seven clusters of ponds, two clusters of ponds with high densities of *An. gambiae *larvae were selected as study sites for the ATSB field trial. Each of these sites included a group of man-made reservoir ponds surrounded by partially flooded rice paddies. The experimental treatment site included six ponds which covered an area of ~3.8 ha and the distance between this group and the closest cluster of ponds was ~2.0 km. The control site included a group of four ponds covering ~1.4 ha which were at least ~0.5 km away from other groups of ponds and 15 to 20 km from the selected treatment site.

### Preparation of ASB and ATSB solutions

The ASB solution included juice of ripe/overripe fruits, 30% Guava juice, 30% Honey Melon juice, 25% water, 12% brown Sugar W/V, 2% local millet beer, and 1% (W/V) BaitStab™ concentrate (Westham, Israel) for preservation and stabilization of the bait. Guava and honey melons were selected for the ASB based on their local availability and their high level of attractiveness for *An. gambiae *s.l. based on comparative field tests in Mali using 26 different types of local fruits (unpublished data). Locally available millet beer was used to start the fermentation process. BaitStab™ is a blend of preservatives and slow-release substances used to preserve food-grade material, and was brought as the only ingredient from Israel [15]. Crushed fruits and the other components were left for two days to ferment in covered plastic buckets in the sun. The liquid, sifted by sieve and then by cloth, was stored at ambient temperature. The pulp was used for goat and chicken feed. ATSB was made by adding the toxin boric acid [24] 1% (W/V) to ASB liquid. Food dye markers of 0.5% W/V Food blue No. 1 or E122, Azorubine, (red) (Stern, Natanya, Israel) were added to ASB and ATSB. A laboratory experiment at University of Bamako confirmed that colonized *An. gambiae *females and males readily fed on the ATSB solution containing food dye marker, most within two hours, and that mortality rates of fed mosquitoes at 12 h were 99.6% (n = 259) for females (2 replicates) and 100% (n = 309) for males (2 replicates) compared to < 1% for control cages of females and males provided only ASB solution.

### Field application of ASB and ATSB solutions

The ASB and ATSB solutions were sprayed with a 16-liter back-pack sprayer (Killaspray, Model 4526, Hozelock, Birmingham UK) in aliquots of ~80 ml on 1 m^2 ^spots at distances of ~3 m on the vegetation around the ponds and rice paddies. Predominant types of plants sprayed at the two sites included rice, sedges, grasses, and non-flowering herbaceous plants. One sprayer completed the applications in less than two hours per site.

### Study design and methods for the ATSB field trial

The field trial was conducted over a period of 38 days, at the end of the malaria transmission season, beginning in mid-November 2008. During this period, adult mosquitoes were sampled a total of 20 times at each site using 6 CDC light traps (Model 512, John W. Hock, Gainesville, FL) without attractants in fixed positions between the ponds. Bait solutions were sprayed on day 8 of the experiment, ASB at the control site and ATSB at the experimental site. The designation of control and experimental treatment sites was done just prior to day 8 based on CDC light trap data showing higher densities of *An. gambiae *s.l. at the experimental treatment site. Collected mosquitoes were sexed and checked for food dye marker using a dissection microscope [13]. They were then preserved in 70% ethanol for species identification by classical taxonomic methods [25] and by PCR to identify species in the *An. gambiae *complex [26]. The physiological age of female mosquitoes was determined by dissecting ovaries and counting the number of dilatations [27]. From the control and treatment sites, live female *An. gambiae *s.l. were randomly selected for age-grading from collections on days 4 and 6 pre-treatment and days 24, 26 and 28 post-treatment.

### Statistical analysis

Statistical analyses were performed with GraphPad Prism 4.0 (GraphPad Software Inc., Calif). Comparisons between light trap catches of female and male *An. gambiae *s.l. at the control and treatment sites were performed using the two-tailed Student's t test. The Z-test was used to compare proportions of female *An. gambiae *s.l. at control and treatment sites that completed more than three gonotrophic cycles. This assessment of "older" females is relevant because gonotrophic cycles of *An. gambiae *are generally three days and the first sporozoites of *P. falciparum *are normally observed in females developing their fourth batch of eggs [28,29].

## Results

PCR analysis showed that two species of the *An. gambiae *complex inhabited the study sites, *An. gambiae *and *An. arabiensis*. *Anopheles gambiae *predominated, and just prior to spraying ASB and ATSB solutions comprised 76.6% (n = 47) and 81.5% (n = 52) of the population in the control and treatment sites, respectively. Post-treatment, the proportion of *An. gambiae *was 81.5% (n = 52) and 100% (n = 54) in the control and treatment sites, respectively.

**Figure 1:**
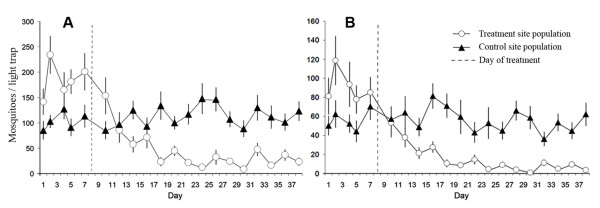
**Relative abundance of *Anopheles gambiae *s. l. females (A) and males (B) in the ATSB-sprayed experimental treatment site and the ASB-sprayed control site, determined by CDC light trap sampling during the 38-day field trial in Bandiagara District, Mali**.

ATSB treatment reduced densities of female and male *An. gambiae *s.l. by about 90%. After spraying ATSB in the treatment site, population densities of female and male *An. gambiae *s.l. declined rapidly over a week and then stabilized at low levels (Figure 1). The pre-treatment of catch 184.6 ± 15.7 females and 55.9 ± 4.7 males per trap decreased to 26.4 ± 3.80 females and 7.35 ± 1.23 males in the last 22 days of the experiment. The control site population was relatively stable yielding pre-treatment levels of 101.6 ± 9.3 females and 55.9 ± 4.66 males per trap, and 118.83 ± 5.82 females and 54.0 ± 3.24 males in the last 22 day of the experiment. A decrease in numbers of mosquitoes collected post-treatment at the ATSB treatment site, when compared with the control site, was highly significant for both females (t = 8.747, df = 13; p < 0.0001) and males (t = 11.91, df = 13; p < 0.0001).

ATSB treatment correspondingly affected the longevity of female *An. gambiae *s.l. as shown in table 1, which summarizes results from the classification of females by age-grading. At the experimental site, most females remaining after ATSB treatment had not completed a single gonotrophic cycle, and only 6% had completed more than three gonotrophic cycles compared with 37% pre-treatment. During the same period at the control site sprayed with ASB (without toxin), the proportion of females completing more than three gonotrophic cycles increased from 28.5% pre-treatment to 47.5% post-treatment.

**Table 1:** Age-group classification of *Anopheles gambiae *s.l. females before and after a single application of ASB (control) or ATSB (experimental treatment) on local vegetation

Site and time	Females examined	% females by observed numbers of dilatations in dissections of ovaries								
		**0**	**1**	**2**	**3**	**4**	**5**	**6**	**7**	**> 8**

Control pre-treatment	200	25.5	23.5	14.5	8	7	4.5	4	3.5	9.5

Control post-treatment	200	12.5	14	16	10	10.5	6.5	9	7	14.5

Experimental pre-treatment	200	23	16.5	12.5	11	8.5	5.5	6	5.5	11.5

Experimental post- treatment	200	52	24.5	9.5	8	3	1.5	0.5	0	1

The difference between the proportion of females with more than 3 gonotropic cycles in the experimental site before and after treatment was significant (z = 7.185; p < 0.05).

At the control site, a high proportion of the *An. gambiae *s.l. females and males fed on the ASB solution sprayed on plants. The proportion of mosquitoes showing evidence of the coloured food dye included in the ASB solution included 56.4% of the females (n = 10,250) and 62.2% of the males (n = 5,071). Even on the last day of collection, 71.1% of the females (n = 741) and 75.1 of the males (n = 373) were marked. A much lower proportion of the mosquitoes contained dye marker at the ATSB treatment site, 3.9% of the females (n = 3,952) and 3.2% of the males (n = 1,325).

## Discussion

The results of this field trial in Mali show that under local conditions a single application of ATSB solution by plant-spraying markedly reduced the relative abundance of *An. gambiae *s.l. populations and their longevity. Within a week after spraying, densities of adult females and males at the treatment site were reduced by around 90% and remained low throughout the remainder of the monitoring period. Clearly, the ATSB treatment was highly effective in killing the "older" more dangerous females as shown in table 1. Reducing the proportion of "older" females is a key factor in reducing malaria transmission [30]. The pronounced impact of the ATSB is comparable to that demonstrated in ATSB field trials in Israel [12-16] and establishes that this method for mosquito control is also highly effective for targeting and killing major malaria vectors in semi-arid areas of Africa.

By using a dye marker in the ASB solution applied at the control site, as in previous studies in Israel [12-16], we demonstrated that a high proportion of the local *An. gambiae *s.l. populations were making contact with and feeding on the solution sprayed on local plants. The observed marking rates for females (56.4%) and males (62.2%), however, represent only minimal rates of contact as the dye marker persists for only about two days due to digestion processes while the mosquitoes can sugar-feed throughout their lifespan. The finding of marked mosquitoes on the last day of collection highlights that the sprayed ASB solution was still present at the very end of the trial. The low percentage (< 5%) of mosquitoes caught with colored ATSB from the treatment site indicates that a high percentage died before again flying where they could be caught, with the possibility that some may have exhibited behavioral changes after feeding on the bait that would have altered their probability of capture [31].

The results demonstrate how ATSB is effective when applied to various types of vegetation located in the vicinity of local mosquito populations, including that which exists around natural larval habitats and is likely used by both newly-emerged and older mosquitoes as outdoor resting sites. This approach is similar to the most recent studies in Israel [16,32] but differs from initial studies of ATSB plant-spraying in Israel where ATSB was selectively sprayed on flowering plants known to be highly attractive to mosquitoes as sugar sources [12,13]. The two approaches are both highly effective and potentially complimentary but the method used in this study and a recent study in Israel [32] is technically simpler as it does not require *a priori *knowledge the most attractive plants. It only requires some basic skills in identifying larval habitats and general types of vegetation that may be used by mosquitoes as outdoor resting sites [32].

The preparation of ATSB solution is technically quite simple. Four of the key ingredients are readily available at the local community level: water, unrefined brown sugar, beer, and ripe/overripe fruit. While initial studies in Israel used nectarines [14,15] and plums in Florida [33], guava and honey melons were used instead based on local availability at the time of our studies and on our comparative tests of the attraction of *An. gambiae *s.l. to various local fruits and seed pods in Mali (unpublished). Even fruits that are close to rotting and are therefore unsuitable for trade and human consumption can be used, and leftover products can be used to feed domestic animals and fowl. As the chemical identity of the attractive ingredients in the fruits has not been determined, at this point it is not possible to substitute a synthesized chemical attractant. Two of the ingredients must be purchased, the BaitStab™ for preservation and stabilization, and the oral toxin, but both are very inexpensive. At the study area in Mali, the boric acid was purchased at the local market.

Rather than using Spinosad ("Tracer™"; Dow Agrosciences, Calgary, Canada) as the oral toxin as in the proof-of-concept studies in Israel [14,15], we instead used boric acid, which is highly lethal to mosquitoes [24,31]. Preliminary laboratory testing in Bamako confirmed high toxicity to *An. gambiae*. The advantage of using boric acid is that it is very inexpensive, readily available, is stable (in contrast to Spinosad which decays by UV), and has a mammalian toxicity level about as low as table salt [34]. The boric acid proved highly effective in our initial field trial. This is not surprising because boric acid and a number of different insecticides have been used for many years as oral toxin for the control of ants, cockroaches, fruit flies, and house flies. Studies by Allan [35] have recently shown that, when delivered orally, a wide variety of different insecticides are effective against mosquitoes, with apparently no repellency effects. The study concluded that baits with oral toxins for mosquitoes using a phagostimulant, such as sucrose, are effective in causing mortality [35]. Longer-term, operational strategies using ATSB solutions with mixtures of 2 or more different insecticides may help minimize the emergence of resistance in local populations of mosquitoes, which is of course already a concern for the insecticides associated with LLIN and IRS use for malaria vector control in Africa [36,37].

This first field trial of ATSB methods in Mali begins to explore some of the ultimate impacts of the ATSB approach for malaria vector control in Africa. In addition to the ATSB plant-spraying methods tested here, it also might be possible to deliver the same ATSB solution using very simple bait stations that have proven successful in Israel [14,15]. Ultimately, we expect that strategies will emerge for co-use of both plant-spraying and bait stations to achieve maximal killing of local vector populations. As the malaria vectors in Africa, *An. gambiae*, *An. funestus *and to a lesser degree *An. arabiensis*, show a pronounced tendency to rest inside houses where they feed on humans [38], it may also be possible to use ATSB methods directly outside or inside houses. Though there were indications of a differential impact on *An. arabiensis *in this trial (i.e., none remained after ATSB treatment), the numbers identified by PCR were too small to detail with certainty that the ATSB treatment had a more pronounced impact on this malaria vector which is well-known to be more exophilic than *An. gambiae*.

Beyond this initial field trial, the full impacts of ATSB need to be determined by field assessments on a larger scale and of longer duration at the village and/or district levels with designs that measure impact not only on vector densities and vector longevity, but also measure malaria parasite transmission (e.g., EIRs), and malaria burden in human populations (e.g., incidence and prevalence of malaria cases). It is also important to determine the additive effects of ATSB when used in combination with existing vector control methods including LLINs and IRS, as it is not likely that ATSB methods alone would be sufficient to meet programmatic goals for malaria vector control. It is also important to determine the full impact on all mosquito species, not just the malaria vectors. In Mali, for example, *Culex quinquefasciatus *is locally abundant and serves as a major nuisance-biting mosquito and a vector of filariasis in some areas [39].

The range of environments in Africa where ATSB methods can be used effectively remains to be determined. They will likely work best in arid and semi-arid areas where natural flowering plants are limited, as the effectiveness of the ATSB methods depends on their ability to outcompete natural plant sources of sugar available to mosquito populations [40]. Though this first field trial was conducted in a semi-arid area of Mali, the actual study sites containing multiple ponds that were highly productive larval habitats surrounded by both natural vegetation and rice paddies were, in general, fairly typical of *An. gambiae *s.l. habitats over a range of environments found in malaria endemic areas of Africa. The ATSB methods may also work well in urban setting and in environmentally altered environments that lack biologically diverse groups of indigenous flowering plants that would naturally sustain mosquito populations. They may also be effective for use in large-scale irrigation areas where rice, for example, is cultivated (rice plants apparently do not provide a source of sucrose for mosquitoes).

There are three further considerations worth noting. First, to optimize performance of ATSB plant-spraying and bait station methods, there is a need to determine the coverage of plant spraying needed and also the density of bait stations needed to achieve effective control. In this first field trial in Mali, spraying just a series of 1 m^2 ^spots of vegetation every 3 m around breeding sites was apparently sufficient. Second, a logistical consideration is that heavy rains will wash off ATSB sprayed on plants and so re-applications during the rainy seasons may be needed. This is one reason why bait stations are equipped with covers [14,15]. During some periods of the year it may be feasible to use both plant-spraying and bait stations but this will depend on local circumstances. Third, ATSB approaches have only minimal risks to humans. Ongoing studies in Israel are determining potential impacts of ATSB on non-target insects. Strategies for spraying ATSB on non-flowering plants may be better than spraying the most attractive flowering plants, in terms of minimizing damage to non-target insects. Honey bees or any of the many species of pollinating bees may be affected and so in Israel suitable metal grids for bait stations have been developed that allow mosquitoes to pass but keep honey bees out (G. Müller and Y. Schlein, unpublished data). Overall, spraying non-flowering vegetation seems to be environmentally safe, except that non-biting midges (Diptera: Chironomidae) feed in similar proportions to the mosquitoes. The ATSB methods may pose only limited environmental risks for the following reasons: 1) whole classes of pollinating insects orient using optical targets rather than scents, 2) beneficial predatory insects will not be harmed because they do not feed on sugar, and 3) pollinating insects are typically absent from some of the prime target areas for ATSB treatment, such as rice fields and areas around mosquito larval habitats where there is minimal flowering vegetation.

In conclusion, this first field trial of ATSB methods in Mali provides a strong indication that such strategies will be very effective for malaria vector control in Africa. If tested further and found to be effective across a range of malaria endemic environments in Africa, it is likely that ATSB approaches could soon be added as a major component of IVM-based malaria vector control programmes. ATSB methods differ from and potentially complement LLIN and IRS methods, which focus on indoor-feeding and resting mosquitoes, because they have so far proven effective in outdoor habitats for killing all physiological states of females and at the same time also kill male mosquitoes. By targeting sugar-feeding mosquitoes in outdoor environments it is likely that their use will on the micro-scale overlap significantly in space with other key life history strategies of malaria vectors including mating and oviposition, both of which are temporally associated with sugar-feeding. Thus, in terms of malaria vector control in Africa, the ATSB methods when used operationally will likely reduce both total numbers of recently emerged female anophelines before they enter houses to feed on humans and the proportion of females exiting houses to oviposit and then returning to houses to re-feed on humans. This strategy to broaden the currently narrow segments of vector populations targeted for control (i.e., those females that feed and rest indoors) is certainly consistent with the broad-based IVM concepts being promoted and implemented throughout Africa.

## Competing interests

The authors declare that they have no competing interests.

## Authors' contributions

GM, YS and JB conceived and planned the study, interpreted results, and wrote the paper. GM directed and performed the field experiments, and analyzed the data. ST and SD facilitated field experiments by selecting study sites and obtaining local clearance from community leaders, and along with MTo, MTr, assisted with the field and laboratory experiments, and data management. All authors read and approved the final manuscript.
